# Sewage intrusion into drinking water distribution systems: implications for water resource management

**DOI:** 10.1007/s10661-025-14607-0

**Published:** 2025-09-26

**Authors:** Jibrin Ndejiko Mohammed, Feroz Mahomed Swalaha

**Affiliations:** Department of Biotechnology and Food Science, Durban University of Technology, P O Box 1334, Durban, South Africa

**Keywords:** Sewage, Intrusion, Water, Distribution, Pathogens, Organic matter

## Abstract

Sewage infiltration of drinking water distribution systems (DWDS) is a major challenge for global water resource management, exacerbated by outdated infrastructure and rising water pressure. According to studies, even small sewage intrusion can result in major microbiological changes, such as appreciable increases in bacterial biomass, bacterial diversity, and dissolved organic matter (DOM) composition. These changes intensify the risk of waterborne infections and complicate treatment procedures required for ensuring safe drinking water. The present work is a review/state of science that synthesizes recent studies on sewage intrusion into the DWDS, focusing on mechanisms and factors that contribute to sewage intrusion, adverse effects of intrusion on water quality, public health, environmental integrity, development and breeding of antibiotic resistance and implications of sewage intrusion into the DWDS for water resource management. The review also explores various strategies for managing and combating sewage intrusion into the DWDS, including infrastructural upgrades, storm water management, improved monitoring systems, regulatory frameworks, and public awareness and engagement programs. To safeguard public health and guarantee the reliability of drinking water supplies in the face of increasing environmental constraints, policymakers and water managers must give priority to investments in resilient infrastructure, innovative treatment techniques, and coordinated measures that address both short-term health issues and long-term sustainability challenges.

## Introduction

The intrusion of waste matter consisting of feces and contaminated water from residents and factories into drinking water distribution systems presents a substantial and rising challenge to universal water resources (Cullmann et al., 2022; Salem et al., 2022). This is exacerbated by several factors, including aging infrastructure, climate transformation, and expanding urbanization. As populations surge and municipalities expand, the demand for clean, potable water keeps increasing. This has placed unprecedented tension on the water distribution systems (Tansel & Zhang, 2022). The networks constructed to distribute safe water become increasingly susceptible to contamination due to physical flaws and operational failures that aid the entry of untreated sewage and impurities. Sewage invasion of drinking water distribution systems can occur via pipe fractures, leaks, longitudinal splits, joint failure, backflows from sewers (Besner et al., 2010; Fan et al., 2024; Tansel & Zhang, 2022), repair works, erratic water supply (Fontanazza et al., 2015) and cross connections (Viñas et al., 2022).

Aging reticulation pipes often corrode and collapse, creating entry points for sewage to infiltrate the distribution system. Studies have indicated that even trivial breakages can cause significant health threats, as pathogenic microorganisms and heavy metals contained in sewage can penetrate water supply networks through such trivial aberrations and rapidly reach thousands of households within hours, thereby posing severe threats to public health (Berg et al., 2023; Fontanazza et al., 2015; Odhiambo et al., 2023; Zeydalinejad et al., 2024). For example, recent research indicated that the intrusion of just 1% sewage into tap water distribution led to dramatic upsurges in bacterial biomass and diversity, as well as a subtle gradient of dissolved organic matter (DOM) that can compromise water treatment measures and compromise water quality (Fan et al., 2024).

Beside compromising treatment measures and water quality, sewage intrusion of drinking water distribution systems (DWDS) has intense and multifaceted public health implications. Polluted drinking water is a well-recognized vector for waterborne infections that can cause substantial health crises, including outbreaks of diarrhoea, cholera, dysentery, cryptosporidiosis, giardiasis, shigellosis, and other illnesses (Fontanazza et al., 2015; Odhiambo et al., 2023; Zeydalinejad et al., 2024). The WHO stated that contaminated drinking water accounts for up to two million annual deaths, including infants and children under the age of five. The financial loss that accompanies polluted water-related health challenges is far-reaching, including excessive costs of treatment that may upsurge during epidemics and decreased production, impacting the local economy (Paraskevopoulos et al., 2024; Wang et al., 2022). Epidemiological reports indicate that shortfalls in water distribution systems showed a relationship with an increased rate of waterborne infections, stressing the need for advanced infrastructural upgrades and intensified monitoring systems (Ercumen et al., 2014; Odhiambo et al., 2023).

Rates of sewage infiltration of drinking water supply networks triggering public health pressures have continuously ravaged the globe. For example, during the COVID-19 plague, Hong Kong had to adopt a sewage monitoring scheme to be able to achieve vital control of the outbreak. The scheme surveyed sewage and wastewater to identify viral waves of COVID-19. This facilitated recognition of infection clusters and assessment of neighbourhood transmission. Though that program was designed to trace COVID-19, it emphasized the potential for sewage to serve as a community-level screening for pathogens’ circulation, accentuating the demand for vigorous monitoring to track epidemics of sewage-related infections (Deng et al., 2022). An investigation in Addis Ababa found *Vibrio cholerae* and *Escherichia coli* O157:H7 in drinking water that posed health threats to the public and implicated open wastewater ditches and defective urban water distribution systems as sources of the pollution (Mogessie et al., 2024). Prior to the Paris 2024 Olympic Games, research stressed the need for wastewater surveillance to identify pathogenic viruses such as poliovirus and influenza viruses. Though this study focuses on avoiding disease outbreaks, it highlights the vital task of examining wastewater systems to alleviate public health threats linked to sewage intrusion of water supply systems (Toro et al., 2024).

**Fig. 1:**
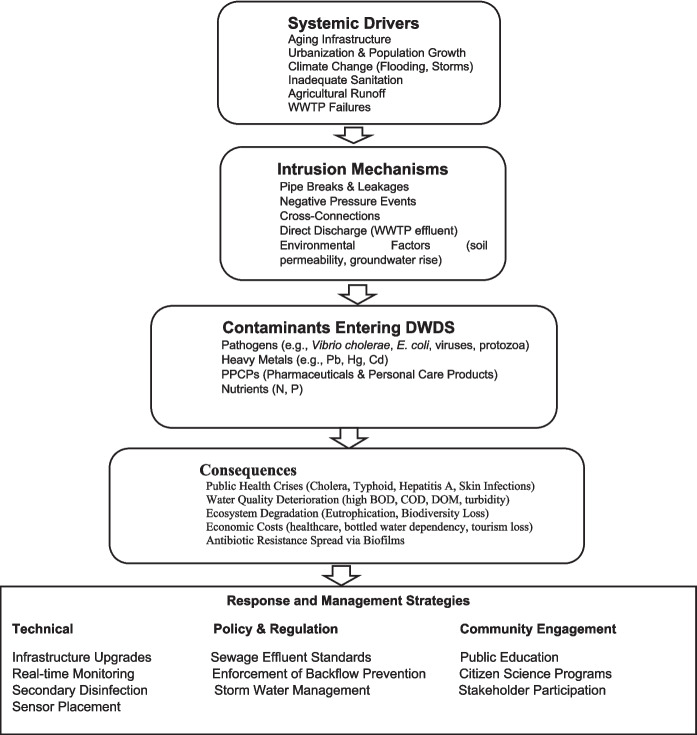
Conceptual framework of sewage intrusion into DWDS: drivers, pathways, impacts and responses

Beside the direct health threats, sewage intrusion has wider economic and social implications as the societies affected by waterborne epidemics face long-term consequences, including reduced property values and increased insurance premiums (Rothenberg et al., 2023). The tension on health care amenities can divert resources from other essential needs, escalating prevailing disparities in access to other amenities (Mohammadi et al., 2024). Furthermore, community confidence in municipal water distribution networks can erode after epidemics that follow incidents of contamination; this can lead to increased dependence on packaged bottled water or unsafe alternate sources that may not be sustainable (Ayanlade, 2024). The present study investigates how a combination of aging infrastructure, hydraulic or structural issues, poor sanitation, and environmental challenges contributes to sewage intrusion into drinking water systems with emphasis on developed nations. It also explores how integrated management strategies can address these overlapping risks to ensure global water safety. The paper focuses on specific components of the water infrastructure that are most vulnerable to contamination. Finally, the study proposes a range of integrated strategies: technical, policy-driven and community-based programs that can effectively mitigate the threats associated with sewage intrusion. These relationships are demonstrated in the conceptual framework summarized in Fig. 1.

### Overview of sewage composition and its impact on human health

Sewage is a complex mixture of several pollutants, including pathogenic microorganisms, organic matter, heavy metals, and other chemicals that can pose significant adverse effects on human health and water treatment procedures. Understanding the complexity of sewage is imperative for creating effective treatment plans and safeguarding the environmental and public health (Hu et al., 2022; Rothenberg et al., 2023). Water constitutes up to 99.9% of sewage, while solids make up around 0.1%, comprising organic and inorganic components that are further grouped into biodegradable materials such as food scraps, human waste, and other components referred to as organic matter (Karri et al., 2021; Vali et al., 2023). The inorganic matter content includes minerals, metals, and non-biodegradable materials. Measurements of biological oxygen demand (BOD), chemical oxygen demand (COD), total suspended solids (TSS), nitrogen, phosphorus, and other elements are used to determine the strength of sewage. For instance, in developing nations, average raw sewage production levels have been estimated to be approximately 50 g/person/day for BOD and 180 g/person/day for total solids (Hu et al., 2022; Xu et al., 2020).

### Pathogenic microorganisms

Pathogens: bacteria, viruses, fungi, and protozoa exist in most sewage water and proliferate at a high rate (Englande Jr et al., 2015; Lin et al., 2022). *Salmonella* spp., *Vibrio cholera,* and *E. coli* are common bacterial pathogens found in sewage. When these organisms infect humans, they cause intestinal illnesses with symptoms like abdominal cramping, diarrhoea, nausea, and vomiting. Sewage harbours viruses such as Hepatitis A and Norovirus that can cause outbreaks of viral gastroenteritis (Berg et al., 2023; Ryu et al., 2019; Teunis et al., 2010). Parasitic protozoans like *Giardia*, *Entamoeba histolytica*, *Entamoeba coli*, *Endolimax nana, Cryptosporidium* spp., and other parasites are often found in sewage. These organisms are especially harmful to people with weakened immune systems and can result in chronic gastrointestinal disorders. *Giardia lamblia* is, for example, a causative protozoan for giardiasis, a duodenal disease characterized by diarrhoea, abdominal pain, and nausea that is one of the prevalent parasites in wastewater samples (Khanum et al., 2012). *Entamoeba histolytica*, which causes amoebic dysentery and is also characterized by severe diarrhoea and abdominal cramps, is isolated along with other parasites in sewage (Khanum et al., 2012) and was reported to be commonly found in environments with poor sanitation (Hamaidi-Chergui et al., 2019). *Entamoeba coli* is an indicator for faecal contamination and the existence of other harmful pathogens; its identification in sewage samples highlights the potential for co-occurrence with other pathogens such as *E. histolytica* (Atabati et al., 2020)*.* Cryptosporidium*,* a waterborne causative parasite of cryptosporidiosis, is characterized by watery diarrhoea and dehydration. This parasite’s oocysts are well known for their resilience in the environment and can resist conventional wastewater treatment procedures, making them a major concern in sewage management (Benito et al., 2020). The intrusion of these parasitic pathogens into DWDS constitutes several challenges in the form of waterborne diseases and public health risks, especially to children, the elderly, and other immunocompromised individuals (Paraskevopoulos et al., 2024; Rothenberg et al., 2023; Teunis et al., 2010).

### Chemical contaminants

Chemical contaminants are commonly found in sewage in the form of heavy metals, organic and inorganic nutrients, pharmaceuticals and personal care products (Karri et al., 2021; Sharaf et al., 2021). Heavy metals such as lead, mercury, cadmium and chromium can be found in sewage, especially those that originate from manufacturing industries (Sharaf et al., 2021). Prolonged exposure to heavy metals can lead to several health issues, including cancer, kidney diseases and neural damage. Hexavalent chromium, for instance, has been indicated in protracted respiratory conditions and carcinogenic illnesses (Lin et al., 2022). Industrial effluents, such as those generated from electroplating, metal fabrication, mining and manufacturing, release wastewater that contains lead (Pb), cadmium (Cd), copper (Cu), zinc (Zn) and mercury (Hg) into metropolitan sewage systems (Karri et al., 2021; Sharaf et al., 2021). Aging plumbing materials made from lead can also leach heavy metals into household wastewater (Karri et al., 2021).

### Nutrients

Nutrients exist in the sewage as nitrogen and phosphorus; their existence in water bodies in excess can cause eutrophication and the formation of hazardous algal blooms (HABs) (Bashir et al., 2020). When humans get exposed to these blooms, the chemicals they generate can harm the liver and cause severe health problems. An ample percentage of nutrients found in sewage originates from anthropogenic waste (Alemayehu et al., 2020). Nitrogen is expelled as urea via excretion, while phosphorus is found in urine and faeces. On average, an individual produces 15 g of nitrogen and 1.5 g of phosphorus in a day via defecation (Albert et al., 2024; Alemayehu et al., 2020). Domestic products such as cleaners and farm inputs such as manures and fertilizers have nitrogen and phosphorus-containing compounds (Karri et al., 2021). For example, laundry detergents and dishwashing liquids contain phosphates that contribute significantly to phosphorus loads in sewage when discharged via the sinks. Leftover fertilizers from farmlands, gardens, and grasslands used or pastured can be washed into sewage systems via runoff waters, contributing to the nutrient contents of the sewage (Fan et al., 2024). Certain food processing industries that deal with organic matter rich in nitrogen and phosphorus may discharge wastewater containing elevated levels of nutrients (Karri et al., 2021).

A major concern for researchers and water managers should be how interactions among microbial groups, nutrient piles, and chemical contaminants in sewage influence the development, tenacity, and environmental behavior of pathogenic and antibiotic-resistant organisms in water distribution system. Answering this question will connect microbiology, environmental chemistry, and public health, examining the *how* and *why* of interactions of these contaminants rather than focusing only on their presence.

### Pharmaceuticals and personal care products

Due to their broad use and the complications in their removal in wastewater treatment processes along with poor monitoring, pharmaceuticals and personal care products (PPCPs) have turned out to be some of the critical environmental pollutants (Caban & Stepnowski, 2021). Serious concerns have emerged about their potential effects on aquatic environments and human health when they find their way into sewage systems. They can interrupt endocrine functions and aid in the development of antimicrobial resistance (Caban & Stepnowski, 2021; Singh et al., 2024; Thulasisingh et al., 2024). When pharmaceutical products find their way into sewage through many pathways such as human excretion, which follows their ingestion and subsequent metabolization, some are defecated unchanged, while others are excreted as metabolites in urine and faeces (Caban & Stepnowski, 2021; Singh et al., 2024; Thulasisingh et al., 2024).

Personal care products like soap, shampoo, ointment, and cosmetics are flushed down the drains during daily routine personal hygiene, also contributing to the PPCP load in the sewage (Singh et al., 2024). Hospitals and clinics gather substantial amounts of pharmacological waste, including unused prescriptions and clinical remains that can find their way into the sewage systems (Thulasisingh et al., 2024). Veterinary drugs applied in livestock farming can also enter sewage through animal wastes, spills, or runoff from farmlands where manure is applied. In fact, about 80% of antibiotic use in the USA is ascribed to agriculture, raising apprehensions about their impact on both human health and the ecosystems (Caban & Stepnowski, 2021; Singh et al., 2024).

PPCPs have been identified in many water bodies, including surface water (Choi et al., 2008; Postigo et al., 2010), groundwater (Choi et al., 2008) and wastewater treatment plant (WWTP) effluents (Bisognin et al., 2021). Their concentrations habitually range from nanograms per liter (ng/L) to micrograms per liter (µg/L). Studies indicate the presence of several pharmaceutical products in drinking water systems at levels that may be detrimental to aquatic life and human health (Albert et al., 2024; Choi et al., 2008; Hu et al., 2022). One serious challenge with PPCPs is their persistence in the environment. Many PPCP complexes cannot be completely removed via conventional sewage treatment, as most standard treatment procedures are intended primarily to remove dissolved organic matter and nutrients rather than specific pollutants like PPCPs (Cizmas et al., 2015; Singh et al., 2024). This allows PPCPs to be discharged into receiving waters or percolate into the groundwater (Thulasisingh et al., 2024).

### Mechanisms and factors that contribute to sewage intrusion into DWDS

To mitigate and prevent sewage intrusion into water supply systems, there is an imperative need to understand the mechanisms underlying their intrusion, with a focus on the conditions that facilitate each of the mechanisms (Englande et al., 2015). Sewage intrusion occurs through several mechanisms, often linked to the physical integrity of the water infrastructure and hydraulic conditions within the system. Although wastewater treatment plants are intended to decontaminate sewage, operational failures during treatment can occur, making it possible for pathogenic organisms and other pollutants to escape into surface water that serves as a major source of drinking water; thus, there is a high risk of polluting the supply system if the WWTPs do not sufficiently eliminate all harmful agents (Lin et al., 2022).

Several studies have proposed different approaches for assessment of the risk of intrusions that occur in water distribution networks and mitigation trials, including optimization studies for identifying critical nodes that need the application of sensors (Berry et al., 2006; Rathi et al., 2015) and statistical-based methods for sensor applications and control measures (Perelman & Ostfeld, 2011). More recently, artificial intelligence is emerging as a useful tool for facilitating sensor monitoring via real-time data analysis, detection of anomalies, and predictive maintenance. AI-driven models can process vast amounts of sensor data to identify early warning signs of contamination, allowing for faster and more targeted interventions. Table 1 summarizes some recent studies on sewage intrusion into drinking water distribution systems. The studies attributed various pollution of drinking water supply systems and groundwater to sewer exfiltration and many other associated causes. Most of the studies investigate explicit factors and employ different methodological approaches to arrive at the outlined major findings. The methods used span GIS analysis to sophisticated simulations and microbiological risk assessment techniques used to offer a thorough grip of sewage intrusion-associated issues.

**Table 1:** Summary of some recent studies on sewage intrusion into drinking water distribution systems

Subject	Factors considered	Methodological approach	Major takeaways from the study	Reference
Circulation of sewer exfiltration to municipal groundwater	Exfiltration rate Aging sewer infrastructure	Geographic information system	With the existing sewer infrastructural replacement frequency, sewer networks pose a risk to urban groundwater	Chisala and Lerner (2008)
Health risks due to sewage intrusion into the drinking water distribution network	Pipe break, inadequate water supply, sustained low pressure, groundwater levels and concentration of pathogens in intruding water	Hydraulic modelling for determining pressure in the DWDS. Pressure level simulation in the water DWDS Simulation of pathogen spatial distribution	The number of pathogenic organisms in intruding water and the extent of the low-pressure events affected the potential for infection more than every other factor	Odhiambo et al. (2023)
Framework for estimation of infection risk from cross-connection and backflow events	- Cross connections - Backflows	- Fault tree analysis - Quantitative microbial risk assessment	The daily cross-connection and backflow-associated infection risk was above the tolerable target level for all reference pathogens and modelled cases	Viñas et al. (2022)
Sanitary sewage overflows (SSO) and boil water advisories (BWA), respectively, contaminate DWDS and generate negative pressures	Negative pressure Sanitary sewage overflow	A symmetric bi-directional case-crossover design was applied to evaluate the role of SSOs and BWAs on emergency room and urgent care visits with a primary diagnosis of GI diseases	SSOs and BWAs were linked to increased emergency room and urgent care diagnoses of GI infections due to sewage intrusion of the DWDS	Rothenberg et al. (2023)
Determination of disruptive effects of sewage intrusion of water distribution systems	Intruded bacteria Dissolved organic matter (DOM) 1% sewage intrusion	Simulation of dynamics in DWDS. Fourier Transform Ion Cyclotron Resonance Mass Spectrometry (FT-ICR MS), total cell counts (TCC), adenosine triphosphate (ATP) and 16S rRNA high-throughput sequencing	Intrusion of 1% sewage into DWDS caused immediate contamination, including an eightfold rise in biomass, a 48.9% surge in bacterial species, a 12.5% upsurge in organic carbon content and a 13.5% rise in DOM	Fan et al. (2024)
Risk model for envisaging incursion and dilution of viruses and their transfer to consumers	Negative pressure Viral load of the soil surrounding the distribution system	Simulation of random entry and dilution of virus through entrenchment of hydraulic model into a Monte Carlo model	The potential for intrusion of drinking water was small, but when this happens, the viral load tends to be high and leads to considerable risk of infection. The presence of chlorine residual reduces the infection risk	Teunis et al. (2010)
Estimation of the protection offered by secondary disinfection in response to an intrusion event	Disinfectant residuals, water quality parameters (pH and temperature), inactivation of microorganisms and intrusion water dilution ratios	Distribution system modelling, inactivation and disinfectant decay modelling and conservative assumptions based on available data	Free chlorine residual (0.5 mg/L) may be insufficient for adequate control of organisms with resistances like Giardia, taking hours to attain disinfection even at low pH and elevated temperatures	Betanzo et al. (2008)
An innovative approach for sensor placement to monitor leaks and pollutant invasion of DWDS	Leakage events Contaminant intrusion	Fuzzy C-means algorithm Generation of leakage and contaminant intrusion events Random integration of sensors to acquire the metric values Evolutionary algorithm (EA) module	The proposed models were superior to other models, and the total number of detections and the detection stabilities were enhanced	Besner et al. (2010)
Modelling health effects of wastewater intrusion into the water distribution system	Organic material degradation Chlorine decay mechanisms Pathogen inactivation kinetics Epileptic water demand	A quantitative microbial risk assessment framework concentrating on selected pathogens	Water contamination events caused infections even with chlorination Concentration and inactivation of pathogens affect infection risk Enterovirus infection risk was higher than Campylobacter* i*n chlorinated systems	Paraskevopoulos et al. (2024)
Assessment of estimated changes in corrosion rates of iron pipes due to incursion of salt water and rise in sea level in coastal areas	Intrusion of salt water Salinity of intruding water Rise in sea level	Assessment of projected shifts in failure rates of iron pipes due to saltwater intrusion and sea level rise in coastal areas	Intrusion of saltwater and rise in sea level can compromise buried pipes Salinity increases groundwater conductivity, leading to an increase in the corrosion rate of iron pipes Dissolved oxygen and chloride ions can expressively increase the corrosion rate	Tansel and Zhang (2022)

The synthesized findings presented in Table 1 underscore that sewage intrusion into water distribution systems represents a critical public health hazard, predominantly driven by microbial contamination and structural or hydraulic vulnerabilities within the network. The works of Odhiambo et al. (2023) and Viñas et al. (2022) show that pipe breaks, low-pressure events, and cross-connections lead to circumstances that favor microbial contamination. Furthermore, the multifaceted effects of aged infrastructure, environmental pressures, and system design-based restrictions can elevate the possibility and severity of contaminations. Chisala and Lerner (2008) and Tansel and Zhang (2022) demonstrated how exfiltration and saltwater incursion, respectively, compromised the structural integrity of pipes, escalating the threat of sewage leakage and corrosion. Meanwhile, other approaches such as sensor optimization (Besner et al., 2010) and advanced disinfection modeling (Betanzo et al., 2008) have been explored to spot and alleviate these threats. Overall, these studies together emphasize a core concern: traditional maintenance and disinfection practices are obviously inadequate, considering evolving environmental and infrastructural conditions. This necessitates more vigorous, adaptive, and pre-emptive water quality management policies.

While the fears raised about the threats of sewage infiltration of water supply systems are valid, the severity of these concerns may be exaggerated when viewed through the lens of ongoing high-tech infrastructural resilience. Many water infrastructural facilities in advanced and progressively evolving regions are implementing multi-barrier tactics, such as real-time monitoring, pressure regulations, and subordinate disinfection (Abd-Elaty et al., 2021; Albert et al., 2024; Alves et al., 2024), which considerably reduce the possibility and effect of contaminations. For example, while some researchers found that 1% sewage intrusion can elevate microbial loads (Fan et al., 2024), such circumstances are infrequent in well-maintained infrastructural systems and lingering disinfectants such as chlorine may mitigate the viability of pathogens prior to exposure events. Moreover, the modeling methods adopted in most of the cited studies are often based on conservative assumptions and simulated worst-case situations, which do not represent characteristic operational veracities. Therefore, while vigilance and incessant upgrades are essential, the data do not generally support a looming or prevalent health crisis from sewage intrusion, predominantly in systems that apply modern risk-based water safety planning.

### Negative pressures and hydraulic failures

Occasional occurrence of low and negative pressures within water distribution networks can possibly lead to pathogen intrusion, especially in the presence of an external source of contamination (such as nearby sewage) and a pathway for the intrusion (leakages in the water distribution system) (Besner et al., 2010, 2011). Negative pressure events in the water distribution networks may bring about a fall below atmospheric pressure, resulting in conditions that let water from surroundings, including sewage, infiltrate the potable water distribution systems (Kauppinen et al., 2019; Mu et al., 2021). Negative pressure events from pipeline disruptions, pump failures, and occasional water demand variations are also capable of reversing the water flow, thereby allowing polluted water from surroundings, including sewage systems, to flow into drinking water pipes (Odhiambo et al., 2023). Pump failure as a result of poor power supply or mechanical failures in pumping stations could also cause a substantial fall in water flow rate; for example, shutting down the pumping systems swiftly can result in a temporal negative pressure in the system (Besner et al., 2010). Similarly, water demand fluctuations, especially during peak usage times, can create imbalance within the distribution system and cause low pressure in certain areas of the distribution system (Abhijith & Ostfeld, 2022; Tansel & Zhang, 2022; Thom et al., 2022).

Nonetheless, the notion that adverse pressure conditions in water supply systems consistently lead to pathogen intrusion remains a subject of debate. This is because all three circumstances; —presence of pollutants, a decline in pressure, and a leak path which—should occur simultaneously, may be rare in well-maintained water distribution systems. Modern water distribution systems with enhanced infrastructure, swift response procedures, and cutting-edge monitoring technologies are usually robust and resilient and can reduce the possibility and impact of pressure-related events. Overall, these views emphasize that while negative pressure poses a theoretical risk, its practical impact on public health can be eliminated if systems are properly managed.

### Ageing infrastructure

The age of infrastructural facilities used for water distribution plays a critical role in the susceptibility of DWDS to sewage intrusion. Ageing and poorly maintained water supply systems often become compromised in terms of their integrity and functionality, eventually making the system vulnerable to contamination by the polluted water. Material fatigue and external pressures generated from soil movement and traffic loads also make older pipes prone to breaks that bring about negative pressure within the DWDS (Islam et al., 2015; Odhiambo et al., 2023). The surrounding soil condition can affect pipe integrity. For example, eroding soils can speed up the degradation of piping materials and thus increase leakage rates that create pathways for sewage intrusion (Odhiambo et al., 2023; Rothenberg et al., 2023).

Leakage and loss of pressure also lead to loss of treated drinking water; for example, Islam et al. (2015) estimated loss of about 22 billion litres (GL) of treated drinking water daily due to poorly maintained distribution systems across the USA and attributed such loss to leakages. Not only does this signify waste of valued water resources but also establish conducive conditions for sewage intrusion. Loss of pressure within the system because of such leaks can cause back siphonage, allowing sewage or polluted groundwater to infiltrate into DWDS (Odhiambo et al., 2023; Westerberg, 2022). Pressure fluctuations associated with the aging systems due to intermittent maintenance and operations can build up negative pressure that enhances the entry of pollutants into the distribution system (Kauppinen et al., 2019; Salehi, 2022). In an attempt to estimate pipe-break and intermittent water supply infection risks, Odhiambo et al. (2023) examined three risk conditions, including pipe-break with no accompanying sewage infiltration, pipe-break accompanied by infiltration, and inadequate water flow through leaking pipes. The researchers used pressure distribution generated from hydraulic models to estimate groundwater heights, and concentrations of pathogens in infiltrating water to estimate the intrusion and number of pathogens in the distribution system. They found daily infection potential to exceed the tolerable target of 10^−6^ for the majority of the water distribution systems and for all scenarios, with the amount of pathogens in the infiltrating water and length of low-pressure instigating events affecting the possibility of infection most. Insufficient maintenance and no monitoring of aging distribution facilities account for unheeded breaks and leakages that can aggravate the threat of sewage intrusion (Palma et al., 2024; Rothenberg et al., 2023). Many municipalities are habitually confronted with economic limitations that interrupt the required infrastructure upgrades and replacements of aging facilities, causing cycles of infrastructural deterioration and increased distribution system pollution (Millington & Scheba, 2021; Rothenberg et al., 2023).

While ageing infrastructure is regularly cited as a key cause of sewage intrusion of the distribution systems, this alone does not necessarily determine system susceptibility. The quality of the system design, management of operations, and maintenance practices can essentially affect the system integrity. Even new-fangled systems can become vulnerable if they are not properly managed, while old ones with proper oversight can function efficiently. High-tech progress, including lining of the pipes internally, monitoring of the pressures in real-time, and smarter grid systems can provide cost-effective substitutes to full-scale infrastructural replacement through enhancement of infrastructural performance and reduction of contamination risks. Overemphasizing infrastructural aging as a major factor that brings about intrusion, as is widely found in the literature, should not divert the attention of the stakeholders from systemic and behavioral issues like late response signals, underskilled workforce, and lack of or inadequate emergency measures that have a more direct impact on water safety.

### Cross-connections

Cross-connection-aided backflow is one of the most severe municipal public health menaces associated with water distribution systems (Viñas et al., 2022). Cross-connections are critical locations where potable water and untreated water may intersect, raising serious concerns about sewage intrusion. Such connections occur due to substandard plumbing practice or deliberate adjustments that do not consider safety procedures (Geldreich, 2020; Salehi, 2022). Global best practices that halt, identify, and eliminate cross-connections and backflows into DWDS have been long established for water resource management to reduce contamination threats. Such measures include monitoring and regulation of new connections and assessment plans for detecting wrongly connected sewage (Abdelgader et al., 2024; Adam et al., 2024; Ayanlade et al., 2024; Islam et al., 2015).

**Fig. 2:**
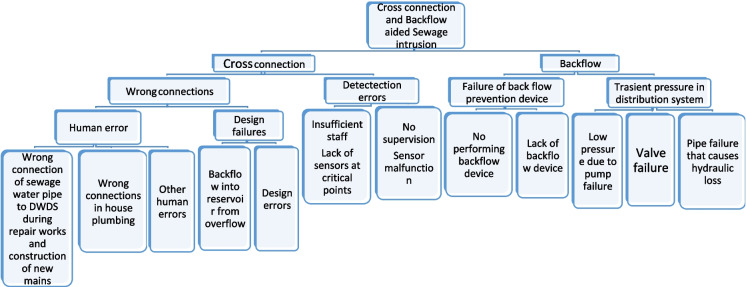
A flow of fault analysis that causes cross-connection and backflow-aided sewage intrusion in DWDS

There are often direct physical connections between the DWDS and polluted water sources, such as sewer systems (Herschan et al., 2020), for example, when a hosepipe is coupled to a spout and a sewer line, or a pipe is connected to the water supply and a drain (Geldreich, 2020; Toprak et al., 2024). They aid potential backflow or back siphonage that draws contaminated water into the potable water distribution systems due to certain conditions, such as the existence of vacuums or pressure differences. This mostly occurs when a water supply line is connected to an improperly or partially vented tank or connected to a plumbing fitting that is underneath a sewer. Backflow occurs when the polluted source’s pressure exceeds that of the clean water distribution system. This allows the polluted water to flow back into the distribution system, while back siphonage occurs because of the vacuum created in the distribution system (Salehi, 2022). Other faults in the distribution system that could cause cross-connection and backflow-facilitated intrusion include wrong connection of a sewage water pipe to DWDS during repair works and construction of new mains, wrong connections in in-house plumbing, design error, sensor malfunctioning, lack of no-performing-performing backflow device and valve failures, as illustrated in Fig. 2.

In order to provide a basis for estimation of infection risk associated with cross-connections and backflows, Viñas et al. (2022) used *Campylobacter, norovirus*, and *Cryptosporidium* as reference pathogenic organisms to construct a hypothetical framework using the fault tree analysis method. The Sweden cross-connection incident data accumulated over a decade and local data from the Gothenburg network were used for determination of the likelihood of a pollution event happening in Swedish water distribution networks and evaluation of the framework, respectively. Their result indicated the daily infection risk arising from backflows and cross-connection events to be generally higher than the acceptable limit of 10^−6^ for the pathogens and the models used, except for the Gothenburg distribution network, which had an infection risk less than 10^−7^. The epidemic case study, simulated via hydraulic model and risk estimation calculated with quantitative microbial risk assessment, predicted between 97 and 148 disease cases characterized with similar symptoms. The fault tree analysis frameworks can be helpful in early microbial risk assessment of water distribution systems (Viñas et al., 2022).

### Environmental conditions mediated sewage intrusion

Environmental conditions such as extreme weather events, soil conditions, groundwater level and seismic activity can cause sewage intrusion and significantly influence the severity of intrusion into DWDS (Li et al., 2024a, 2024b; Toprak et al., 2024). Extreme weather events like heavy rains and flooding can overwhelm the sewer systems and cause overflows. Hurricanes and tropical storms are capable of damaging distribution infrastructure, causing leaks and breaks in the pipes. Seismic events, such as earthquakes, can also damage distribution infrastructure and allow heavy intrusion into the distribution systems (Li et al., 2024a, 2024b; Liu et al., 2020; Toprak et al., 2024).

### Direct discharge from WWTP-aided intrusion

The quantity and intricacy of wastewater have continued to grow in response to an increase in municipal populations and the industrial revolution, necessitating effective treatment and innovative management methods. Though WWTPs are meant for the treatment of sewage, inadequacies in the treatment protocols can result in pollution of the receiving water bodies and the environment (Ahmed et al., 2022; Benito et al., 2020). The presence of harmful contaminants in industry-generated wastewater poses significant challenges during the treatment, including disruption of biological activities that are necessary for efficient wastewater treatment and suboptimal effluent quality. Sewage invasion arising from direct discharge from treatment plants is nowadays rampant and acknowledged as a serious environmental challenge in any region where effluents from treatment plants are frequently spilled. Though the Clean Water Act (CWA) necessitates toxicity tests prior to the discharge of WWTPs’ effluent to establish the safety of the effluent, more investigations may also be required to figure out what modifications to treatment procedures are needed (Benito et al., 2020).

Studies have also shown that secondary and tertiary treatments do not completely eliminate some bacteria, which could cause health threats when effluent from such treatment gets released into natural waters or is repurposed for irrigation or other uses (Ahmed et al., 2022; Englande Jr. et al., 2015). The treated effluent may harbour excessive nutrients in forms of phosphates and nitrogen; these nutrients have the potential to cause eutrophication in the receiving water, leading to algal bloom formation that lowers oxygen content of the water and disturbs aquatic ecosystems. The resultant degradation of water quality may facilitate seeping of polluted surface water into groundwater supplies, reaching the sources of drinking water (Benito et al., 2020). Similarly, organic contaminants and residual heavy metals from industrial discharges may still be present in treated wastewater. These materials can build up in sediments and may not completely be eliminated during treatment procedures (Alves et al., 2024).

Waste water treatment plants may release untreated or insufficiently treated wastewater into the environment when they do not meet regulatory standards for effluent quality (Benito et al., 2020). For instance, contaminants like heavy metals, minerals, and pathogens can seep into groundwater and surface waters, eventually finding their way into drinking water supplies (Englande Jr. et al., 2015). Facilities may release untreated sewage straight into rivers in situations of extreme non-compliance. Because it contaminates sources of drinking water, this not only damages aquatic ecosystems but also directly endangers human health (Bisognin et al., 2021). Although such operations may face fines or legal action from regulatory bodies, the immediate environmental damage is still a major concern. Strict adherence to regulatory requirements is necessary to address these problems (Bisognin et al., 2021).

### Agricultural practices

One of the main causes of drinking water supply contamination is agricultural practices. Agricultural pollutants can find their way into the water distribution system via nutrient runoff, pesticide use, livestock waste management, improper waste disposal and other detrimental chemicals (Karri et al., 2021). Rainfall and irrigation events often aid the washing of nutrients, particularly nitrogen and phosphorus, into the nearby water bodies. High concentrations of such nutrients in the surface water can lead to eutrophication and the accompanying formation of algal blooms, thereby reducing the water quality and constituting public health hazards when such water is used as a source of drinking water (Al-Hazmi et al., 2023; Zahoor & Mushtaq, 2023).

Substantial amounts of manure are accumulated via intensive animal farming (Zahoor & Mushtaq, 2023). Water bodies may become directly contaminated because of improper disposal of agricultural waste, including improperly treated or untreated sewage from neighboring communities or farm activities. This is especially troublesome in places where farming is concentrated close to supplies of potable water. Sedimentation in adjacent water bodies can be exacerbated by agricultural operations that cause soil erosion (Wang et al., 2022; Zahoor & Mushtaq, 2023). Water quality can be further deteriorated by sediments carrying pollutants like pesticides and nutrients. Additionally, this sediment-rich flow can choke drinking water treatment plants, making the process more difficult. To mitigate agricultural practices-related intrusions, it is crucial to implement sustainable agricultural practices, enhance regulatory frameworks, and promote effective waste management strategies that protect both water quality and public health.

The mechanisms and factors that contribute to sewage infiltration into DWDS interact in an intricate, mutually dependent manner that increases vulnerability across the infrastructure, environmental, and operating realms. Ageing infrastructure, accompanied by corrosion or deterioration of pipes, serves as a prime enabler through the creation of physical entry paths such as cracks and joint failures, particularly when the system faces dynamic pressures or soil movements. These weaknesses become compounded when negative pressure occasions prompted by pump failures, pipe breaks, or abrupt fluctuations in demand for water, which reverse water movement and draw in contaminants via existing leaks. Cross-connections due to poor plumbing or maintenance-related mistakes, additionally exacerbate adulteration threats by letting crude or untreated water from sewers or storm drains combine with potable supplies, mostly in the absence of a backflow inhibition system or its malfunctioning. Environmental conditions like flooding, rising groundwater heights or soil penetrability escalate this situation through the saturation of the nearby environment with sewage-laden water, heightening the likelihood of infiltration through structurally compromised parts of the system. Direct discharges from encumbered or flopping WWTPs into surface waters, used as drinking water sources, add extra pathogens and other pollutants to the system, particularly source waters with weaker protection. Agricultural activities, such as the use of manure and fertilizers close to water sources, add loads of nutrients and pathogens to the environment. These nutrients and pathogens may intrude DWDS via runoffs and infiltrations. Together, these factors do not impact the water distribution system individually but converge, where, for instance, floodwater overwhelms sewer systems, especially for CSO systems, which may increase contaminant concentrations, adversely impact treatment performance, and stress ageing infrastructure, signifying how multiple factors can synergistically work together to compromise water quality and jeopardize public health.

### Effects of sewage intrusion on ecosystem

The quality of water as well as the nearby environments is endangered by sewage incursions of water supply systems. The weakening water infrastructure and increase in water stress are exacerbating this phenomenon and the accompanying long-term and short-term effects. Sewage intrusion presents multidimensional issues that reduce water quality and upset the ecosystem (Englande Jr. et al., 2015; Kauppinen et al., 2019). Abrupt upsurges in microbial activity and organic content can lead to long-term health risks and environmental degradation. A research report indicated that even a 1% sewage invasion of drinking water can lead to up to an eightfold increase in total bacterial biomass, about a 48.9% rise in the total bacterial species (diversity change), and a 12.5% increase in dissolved organic carbon (DOC) and a 13.5% increase in distinctive dissolved organic matter (Fan et al., 2024). These changes show a shift from a dissolved organic matter-dependent microbial community to a bacterial-governed interactive community, emphasizing the vibrant nature of microbial ecology in the sewage-intruded water supply system (Fan et al., 2024).

Major consequences of sewage intrusion into DWDS that could occur immediately include microbial contamination and chemical contamination. In 2016, following a pipe failure in Finland, wastewater was able to intrude into the DWDS through a maintenance well that had air release valves for both drinking water and wastewater. About 790 people were affected with diarrhoea and stomach pain by the drinking water contamination that resulted from this incident. This incident caused major cross-contamination before detection and maintenance efforts were initiated, and the presence of sapovirus in contaminated drinking water caused about 450 reported illnesses (Bashir et al., 2020; Kauppinen et al., 2019). Polluted water can harbour pathogenic microbial strains that cause diseases like cholera, dysentery, and Hepatitis A, posing severe health burdens to communities that count on these water sources (Albert et al., 2024; Karri et al., 2021; Mapingure et al., 2024). Intrusion of sewage into drinking water distribution has been established to be the lead cause of drinking water adulteration. Heavy metals, nitrates, and other chemical compounds find their way into drinking water via sewage. These scums modify the water flavour and odour in addition to posing health threats. Chlorides and trace metals derived from sewage are responsible for unpleasant tastes and odours often experienced in water (Fan et al., 2024).

The long-standing consequence of sewage incursion extends beyond human health to comprise eutrophication, loss of biodiversity, and microbiological community modification (Albert et al., 2024)**.** Eutrophication ensues when** s**ewage-linked nutrient accumulation causes formation of algal bloom in water bodies. This occurrence reduces the oxygen content of the water, causing “dead zones” with heavy contamination and loss of aquatic life, especially fish and amphibians that are delicate species and can thus be endangered by the contaminated water (Kauppinen et al., 2019). According to Fan et al. (2024), elevated sewage-accumulated nutrients can interfere with reproduction rounds of certain fishes to cause population diminutions of the affected fish species. Changes in microbial dynamics of the aquatic environments may eliminate the beneficial microorganisms needed for preservation of the ecosystem and promote the growth of dangerous species (Albert et al., 2024; Karri et al., 2021).

Protracted contact with certain pollutants, such as nitrates, can intensify the threat of long-term health challenges. Studies have established a relationship between elevated nitrate levels in drinking water and an increased risk of colorectal cancer. The cancer-causing potential of nitrate was apparent even at quantities lower than stated standards for drinking water (Lin et al., 2022). Neural disarrays that led to delayed development and cognitive deficiencies in children (M. Li et al., 2024a, 2024b; Pavone et al., 2020) have been linked to the presence of heavy metals such as lead that could be found in water supply systems infiltrated by industrial sewage (Sharaf et al., 2021). Sewage incursion of DWDS is thus a complex problem that affects ecosystems and water quality.

### Skin infections

Sewage-contaminated water can cause several skin infections and skin irritations. These infections arise from exposure to pathogenic organisms found in contaminated water or poorly treated sewage. Pathogens such as bacteria and fungi in the skin barrier initiate infections such as cellulitis, dermatitis and more severe soft tissue infections. The risk is especially high for persons who participate in recreational events in sewage-intruded waters or those exposed occupationally to sewage. Skin and soft tissue infections arising from water contamination of wounds involve organisms with uncommon antibiotic resistance patterns and call for a nuanced and directed diagnostic tactic with an adaptation of the usual antibacterial or antifungal choice to accomplish a successful cure, alongside belligerent debridement and wound care (Naidoo et al., 2023).

*Staphylococcus aureus* and *Streptococcus aeromonas s*pecies, particularly *Aeromonas hydrophilia*, have also been indicated in the rapid onset of wound infections that follow exposure to fresh or brackish water (Naidoo et al., 2023). Vibrio species are found in warmed coastal waters and can cause skin infections in individuals exposed to contaminated seawater. Contact with sewage-infiltrated water has also been linked to parasitic diseases such as the ones caused by helminths, including *Schistosoma* (Hamaidi-Chergui et al., 2019), which can penetrate the skin to cause schistosomiasis—a disease characterized by dermatitis and other systemic symptoms (Khanum et al., 2012; Naidoo et al., 2023). Studies have indicated that swimmers exposed to elevated levels of bacteria in recreational waters experience greater rates of skin rashes and infections than non-swimmers (Lin et al., 2022).

Hepatitis A and adenovirus are both transmitted through the faecal-oral route, frequently via polluted drinking or recreational water (McKee & Cruz, 2021). In areas with deprived sanitation, epidemics can arise when individuals get exposed to polluted water sources (Ryu et al., 2019). Abdominal discomfort and jaundice are symptoms of hepatitis A, and conjunctivitis (pink eye) and respiratory infections are symptoms of adenovirus, but skin manifestations such as rashes can also occur due to the systemic infection of both viruses (Lanrewaju et al., 2022; McKee & Cruz, 2021). In addition to its well-known ability to cause gastroenteritis, norovirus, transmitted through direct contact with contaminated persons and contaminated recreational water, can occasionally result in skin rashes. Enteroviruses, including coxsackie viruses and echoviruses that cause hand-foot-and-mouth disease, are characterized by skin lesions and rashes and are also frequently found in contaminated waters (Lanrewaju et al., 2022).

Epidemiological reports obtained from Gaza attributed a significant surge in hepatitis A cases and skin diseases to a severely compromised drinking water supply system degenerated by conflict conditions. Children and the elderly population with suppressed immune systems, professional workers, recreational swimmers and those with pre-existing skin diseases were mainly affected (Shahvisi, 2024). Epidemiological reports also indicated detection of enteric viruses like norovirus and rotavirus in recreational waters and drinking water supply systems that do not meet safety standards (Lanrewaju et al., 2022). Through a variety of routes, such as direct exposure through tainted water sources or indirect exposure through food produced in contaminated soils, the many pollutants present in sewage offer major health concerns to humans.

### Sewage intrusion into drinking water distribution and antibiotic resistance

The sources of antibiotics in sewage include human waste, pharmaceutical discharges, clinical waste, pesticides, and laundry waste. The role of sewage in the development and breeding of antimicrobial resistance is well recognized in the literature. The antibiotic-resistant bacteria (ARB) that have endured a number of antimicrobial agents in sewage and acquired more antibiotic resistance genes (ARGs) can infect humans and animals when sewage invades natural water bodies or DWDS; these constitute a serious danger to public health (Zhuang et al., 2021). However, the view of pesticides and laundry waste as one of antibiotics is less supported in the literature. Pesticides may harbour biocides but cannot be characteristically categorized as antibiotic sources. Likewise, waste generated from laundry may comprise bits of antimicrobial agents accumulated from treated textiles or disinfectants, but there is a paucity of empirical data that it constitutes a major antibiotic input. Thus, their role may be overstated or context-dependent.

The major pathways through which antibiotics get into sewage systems are human waste and improper disposal of pharmaceutical products. An enormous proportion of administered antibiotics that are not absorbed and are defecated unchanged contributes to high concentrations of antibiotics in wastewater (Uluseker et al., 2021). Antibiotic resistance in sewage systems is also driven by wastewater from clinical origins and agricultural runoff, which contain elevated levels of antibiotic-resistant bacteria (ARB) and antibiotic resistance genes (ARGs) (Uluseker et al., 2021). Hospital-generated wastewater was reported to exhibit concentrations of ARGs that are at low concentrations. Likewise, WWTPS are well recognized hotspots for horizontal gene transfer that can facilitate dissemination of resistance genes in bacterial populations (Mortensen et al., 2024; Uluseker et al., 2021; Zhuang et al., 2021). While several researchers (Albert et al., 2024; Besner et al., 2011; Cizmas et al., 2015; Mortensen et al., 2024; Postigo et al., 2010) found sewage as an antibiotic reservoir, the leap from environmental ARGs to human infection necessitates more mechanistic clarity, particularly vis-à-vis their transferability and clinical significance.

Biofilms encompass closely associated bacterial populations that can grow to 10^8^ to 10^11^ bacterial cells per gram of biofilm dry weight (Medina et al., 2020). Such mass collection of microorganisms enables close interfaces among the microbial cells and increases the chance of hereditary interchange, including horizontal gene transfer, which is the primary mechanism for the acquisition to exist, promoting the resilience of the community against the antimicrobials (Al-Hazmi et al., 2023; Medina et al., 2020). The existence of biofilms in wastewater systems offers a perfect dwelling for ARB; they persist and spread resistance genes to other bacteria. The difficulties in removing biofilms further complicate the management of antibiotic resistance (Flores-Vargas et al., 2021). The biofilm structure contains some protective extracellular polymer matrix that creates a barrier for the dissemination of antimicrobials. This matrix significantly lessens the efficiency of antimicrobial treatments and makes biofilm bacteria about a thousand times more resilient than their free-living counterparts (Flores-Vargas et al., 2021; Medina et al., 2020). Thus, bacterial cells within biofilms can survive and persist in the presence of antibiotics. While WWTPs contain different microbial groups advantageous to gene interchange, the occurrence and public health significance of HGT in such situations remain contested. Dilution, environmental stressors and bacterial viability post-treatment can meaningfully affect gene transfer potential.

As the biofilm grows, it gets progressively more difficult to control the spread of antimicrobial resistance. Resistant cells released from biofilm can infiltrate new sites, such as natural waters, thereby disseminating resistance to the ecosystems (Flores-Vargas et al., 2021). Due to their distinct qualities, which include high bacterial density, protective structures, antibiotic accumulation, improved gene transfer capacities, and environmental tolerance, biofilms in sewage systems are important hotspots for the development and spread of antibiotic resistance.

### Impact of intrusion of different sewage types on water distribution systems

Table 2 gives a relative analysis of sewage types and how they impact water distribution networks, highlighting significant public health and infrastructural implications. Few studies, such as those conducted by Teunis et al. (2010) and Pelekanos et al. (2021), stimulated sewage water and applied refined modelling tools, including Monte Carlo simulation tools and EPANET-MSX to evaluate contamination threats and microbial behaviour. Such methods permit a controlled assessment of pathogenic concentrations and their spatial dispersal, indicating that even lower probability infiltration events can cause significant threats of infection, arising from viral and bacterial proliferation. Their findings emphasize the limitations of traditional disinfection practices, as high microbial loads can resist chlorination and continue to proliferate, heightening the health risk to consumers.

**Table 2:** Impact of intrusion of different sewage types on water distribution system synthesized from selected studies

Source/type of sewage	Composition	Methods used for the study	Impact on water distribution system	Reference
Simulated sewage	Organic matter,;viruses	Entrenchment of hydraulic model into a Monte Carlo simulation	Low probability of contamination High viral concentration with considerable infection risk Heterogeneous spatial distribution of infection risk	Teunis et al. (2010)
Contaminated ground water	Reference pathogens (*Campylobacter*, *Cryptosporidium*, norovirus)	Quantitative microbial risk assessment (QMRA) Health risk assessment	Caused acute gastrointestinal illness Daily infection risks above acceptable risk of 10^−6^	Odhiambo et al. (2023)
Sewage from open drainage channels	Pathogens, suspended solids, heavy metals mainly Pb, Cd, Cr, Zn and Cu	SewerCAD software analysis and water quality sampling	Contamination of water sources; increased health risks for local communities	Kinuthia et al. (2020); Liu et al. (2018)
Combined sewer overflow (CSO) during wet weather	Rainwater mixed with wastewater; pathogens, nutrients and chemicals	Infrastructure assessment and regulator analysis	Caused contamination and reduced treatment plant efficiency	Palma et al. (2024)
Saltwater intrusion and sea level rise	Salt water	Mathematical models for simulation of saltwater intrusion following diffusive interface Biscayne aquifer’s models	Intrusion reached 271 m Aquifer salinity reduced up to 11.74%	Abd-Elaty et al. (2021)
Domestic sewage	Pathogens, nutrients (nitrogen and phosphorus), heavy metals and suspended solids	Physicochemical analysis Microbial profile determination qPCR and high-throughput sequencing	Consumes the chlorine disinfectant High contamination caused degradation of water quality	Hu et al. (2023)
Simulated organic sewage	Total organic compound Bacterial loads	Bacterial regrowth model and first parallel chlorine decay model Modelled using EPANET-MSX	Increased bacterial populations that spread across the distribution network Increased health risk to consumers	Pelekanos et al. (2021)
Storm overflow discharge into coastal waters	Bacteria (e.g., *E. coli*), chemicals, fertilizers and pesticides	Enrichment evaluation and fuzzy technique;gridded surface subsurface hydrologic analysis model	High bacteria levels causing beach closures; economic losses due to reduced tourism; algal blooms harming aquatic life	Abbasi et al. (2021)
Industry-based sewage generated from factories	Heavy metals (e.g., lead and mercury), organic pollutants (e.g., solvents)	Industrial effluent monitoring and chemical analysis	Pollution of water sources Health risk for humans and wildlife from heavy metal accumulated in drinking water systems	Shang et al. (2008)
Simulated sewage intrusion due to ageing infrastructure	Dissolved organic matter and pathogenic bacterial	Molecular-level analysis of microbial succession and organic transformation in drinking water systems post-intrusion (1% sewage intrusion)	An eightfold increase in biomass (TCC) Extended commotion of microbiological balance in water systems 48.9% increase in bacterial species diversity Organic carbon content (DOC) increase by 12.5% after intrusion	Fan et al. (2024)
Sanitary sewage overflows	Organic matter, nutrient, suspended solids and wastewater effluents	Symmetric bi-directional case-crossover study design	Sewage contains pathogens, which cause gastrointestinal (GI) infections 13% increase in the odds of a diagnosis for GI infections	Rothenberg et al. (2023)
Model wastewater intrusion events	Organic compounds and enterovirus, *Campylobacter* and *Cryptosporidium*	Quantitative Microbial Risk Assessment framework that focused on enterovirus, *Campylobacter* and *Cryptosporidium* and their dose-responses	Water contamination events caused infections even with chlorination Concentration and inactivation of pathogens affect infection risk Enterovirus infection risk was higher than *Campylobacter* in chlorinated systems	Paraskevopoulos et al. (2024)

In contrast, field-based evaluation of crude sewage, combined sewer overflows (CSOs) and industrially generated sewage contains an extensive spectrum of pollutants, such as nutrients, heavy metals, and suspended solids. Investigation conducted by Hu et al. (2023) and Bhawan (2013) shows that crude domestic sewage can introduce more organic compounds and microbial loads into water treatment networks, possibly overwhelming their capability and compromising their treatment efficiency. Furthermore, industrial sewage, as reported by Shang et al. (2008), harbors perilous pollutants including lead and mercury that can pose chronic health risks and bioaccumulation in the environment. The compounded effect of CSOs during wet weather (Palma et al., 2024) worsens these challenges, indicating how infrastructure inadequacies can cause operational failures and water contamination, especially at peak flow conditions.

Other studies examine the impact of saltwater intrusion and infrastructure ageing on the distribution system. Abd-Elaty et al. (2021) for instance, demonstrated the salinization of freshwater aquifers due to sea-level rise through mathematical modelling, presenting a long-term threat to drinking water resource management. These studies highlight the importance of integrating predictive models, microbial risk assessment, and environmental monitoring into water distribution infrastructures. Generally, confronting sewage meddling into DWDS demands integration of a number of management measures, including regular infrastructural elevations, proper regulations, public participation, and technical innovations, as discussed in the following sections.

## Strategies for managing and combating sewage intrusion into the DWDS

### Infrastructural upgrades

Capital investment in the transformation and modernization of drinking water distribution and wastewater management infrastructures is critical to protecting public health and the environment against sewage intrusion-related threats. Sewer separation and replacement of aged infrastructure remain two major ways of boosting water infrastructure. Piping material type and durability also exert enormous influence on water distribution’s system lifespan and integrity (Lin et al., 2022). Replacing aging pipes with stronger pipes of ductile iron or PVC has the potential to lessen leaks and cracks that make way for sewage incursion (Mapingure et al., 2024; Medina et al., 2020). Biofilm-development-prone to experience breaks; regular valuations and timely substitutions can curtail these risks (O'Keeffe et al., 2024; Qamar et al., 2018).

Water distribution infrastructure systems should be designed or constantly upgraded to resist any pressure surge that can occur during heavy rainfalls or sudden spikes in demand (Qamar et al., 2018). The upgrades and designs should incorporate pressure-regulators that help to maintain steady force and lessen backflow-related perils from the sewers. Preservation of the infrastructure should include repetitive maintenance schedules of water mains and related infrastructure to ensure timely fixing of possible vulnerabilities. This should embrace regular inspection, cleaning of pipes, biofilm eliminations, and checking for signs of corrosion or damage (Abhijith & Ostfeld, 2022; Vali et al., 2023). Twofold water systems that create separate systems for sewage and drinking water, particularly in counties where sewage incursions are common, can drastically reduce contamination risks. Infrastructural upgrades should not be limited to existing systems but also include financing in new infrastructure that can prevent cross-connections between drinking water supplies and sewage lines (Wang et al., 2022).

### Storm water management

Management of storm water is a critical means of combating sewage infiltration of DWDS (Abhijith & Ostfeld, 2022; Alves et al., 2024). This can be accomplished through green infrastructure (GI) solutions such as the creation of rain gardens, permeable pavements and bioswales that can mimic natural processes in terms of absorbing and filtering storm water. Regulatory incentives in the form of fee discounts and grants for the implementation of GI systems can motivate municipalities and developers to apply such sustainable practices (Alves et al., 2024; Farooq et al., 2022). By integrating green infrastructure with the traditional infrastructural schemes, cosmopolitans can uphold resilience against floods, thus ensuring cost-effective protection of water quality. A well-structured storm water management strategy should have the components outlined below.

1. Public enlightenment and outreach programs notify the public on possible public health threats associated with storm water and effective mitigations.
2. Illicit discharge identification and removal protocol identify and remove illegal discharge into storm water paths.
3. Construction of runoff controls adopts measures for handling runoffs accumulated from construction sites.
4. Post-construction of runoff controls guarantees efficient management of storm water after construction.
5. Maintenance and inspection regularly maintain storm water facilities to combat blockages and ensure functionality.

### Regulatory frameworks

A vigorous and dynamic regulation for combating sewage intrusion of DWDS should integrate higher treatment practices and ensure regular compliance. An advanced treatment and standard protocol will mandate sewage treatment technology that ensures effluents meet standard criteria stringently prior to their discharge. This will minimize the entry of pollutants into water bodies. Specific enhanced treatment standards that incorporate advanced filtration systems, disinfection protocols, nutrient removal and source control measures can enhance the overall quality of the treated wastewater before it is discharged into the environment (Abhijith & Ostfeld, 2022; Alves et al., 2024).

Monitoring measures should cover regular sampling and evaluation of water quality with an emphasis on pollutant detection and compliance with laid-out standards; these must be updated and documented. Corrective measures and penalties must be implemented where standards are not met. This approach can help to mitigate sewage overflow-associated risks and further protect public health.

### Public awareness and engagement

Public enlightenments and engagements are essential for combating sewage incursion of DWDS. These strategies should focus on communal programs, citizen science schemes, and public outreach creativities (Abhijith & Ostfeld, 2022; Nadeem et al., 2023). They could take forms of workshops, seminars, and campaigns facilitated by experts and entertainers who can create excellent platforms for enlightening populaces on the significance of appropriate sewage management and how inappropriate sewage disposal can influence public health and the environment (Farooq et al., 2022; Nadeem et al., 2023). Such programs should focus on schools, markets and local organizations to build a comprehensive understanding of risks and issues around the sewage.

Citizen science projects that involve capable community members in evaluating local water quality can motivate community members to act against bad sewage management (Hegarty et al., 2021). Such schemes should involve teaching the volunteers how to collect water samples and how to report incidences of intrusion. This can promote local stewardship and responsibility among the members of the community (Hegarty et al., 2021; Nadeem et al., 2023). Community outreach creativities that employ social media and community broadcast apparatuses can help to spread awareness on effective wastewater management procedures and boost responsible waste disposal (Hegarty et al., 2021). These strategic measures not only edify the public but also encourage public ownership of water resource management, leading to better practices and reducing sewage invasion of the water systems (Mahajna et al., 2022).

### Monitoring programs

A vital element of an integrative sewage incursion control mechanism is the application of an innovative monitoring facility that permits real-time assessment of water quality and inherent pressure within the distribution system (Mahajna et al., 2022). This enables the swift and effectual discovery of anomalies, including sudden decreases in pressure and changes in the population of the microorganisms that indicate sewage intrusion. An encircling perception of water system function and support at the anticipated failure points can be achieved through integration of data generation using sensors entrenched in the distribution systems (Abhijith & Ostfeld, 2022; Hegarty et al., 2021). This preemptive approach is critical for preserving water quality and swift response to contamination events. The following measures are recommended for monitoring plans to ensure effective tracking of sewage intrusion of the water supply system:

1. Real-time assessment strategies that adopt flow meters and sensors to persistently measure water peaks and flow rates in sewer systems allow prompt detection of overflows and incongruities.
2. Data incorporation that implements geographic information systems (GIS) to view and assess data facilitates decision-making on management of sewage and infrastructural needs.
3. Pathogen surveillance that determines public health hazards through quantitative microbial risk assessment (QMRA) frameworks.
4. Remote access and cloud computing that apply cloud platforms for storing and analysing data allow remote monitoring and rapid response to sewage incursions.
5. Consistent repairs and standardization to guarantee regular maintenance and calibration of the monitoring facilities ensure accurate and reliable collection of data (Abhijith & Ostfeld, 2022; Mahajna et al., 2022). These strategies mutually boost the capacity to effectively detect and respond to sewage infiltration.

## Conclusion

Sewage intrusion into the water distribution network system is a critical issue with a dire implication for management of global water resources, necessitating prompt and incessant attention and action. Research explorations implicated obsolete infrastructure and growing stress on the water distribution system triggered by increasing urbanization and climate alteration, as the topmost cause of the systems’ susceptibility. The mechanisms/factors that contribute to sewage intrusion of DWDS include negative pressures and hydraulic failures, aging infrastructure, cross-connections, environmental conditions, discharge from WWTPs, and agricultural practices. The immediate health risks associated with sewage intrusion are frequently and well known, but the long-term impacts on the purity of water and microbiological underlying forces remain less clear and alarming. Based on research findings, even minuscule intrusion can inflict a significant alteration on the microbial community of the water, with consequences of up to eight-fold increase in biomass and a substantial upsurge in bacterial diversity. These changes do not only pose health risks but complicate water purification management, as the traditional management or purification methods may not adequately tackle the transformed microbiological condition of the water. The persistence of DOM in the water and water distribution system and peculiar hurdles of water treatment procedures increase the cost of water management, stalling compliance with regulations aimed at safeguarding water supply systems. The implications extend beyond direct health threats; they further underscore the requirement for an integrated strategy that reflects the interdependence of ecological health, sanitation, and water supply systems. These intricacies are frequently not well addressed by prevailing regulatory frameworks, as disjointed methods that ineffectively decrease the hazards associated with sewage intrusion. Stakeholders need to prioritize funding of infrastructural resilience that embraces advanced monitoring systems and encourages rigorous state-of-the-art models to guarantee safe and dependable potable water for even future generations as global populaces continue to increase and climate change excavates. To enhance resilience against fears of contamination, it is essential to adopt an all-inclusive position that considers infrastructure upgrading, real-time monitoring machines, and community participation in water resource management. Future investigations must lay emphasis on longitudinal studies of waterborne disease epidemics related to pressure loss incidents in urban water distribution systems. Further investigations should also focus critically on feasibility or trade-offs that weigh different solutions such as real-time monitoring versus periodic assessments, use of chlorine disinfection versus other disinfection. Besides, cost-merit analyses of pre-emptive maintenance against reactive outbreak retorts could provide guidance and priority for international investment.

## Data Availability

No datasets were generated or analysed during the current study.
